# A low-frequency multiple-band sound insulator without blocking ventilation along a pipe

**DOI:** 10.1038/s41598-022-21673-8

**Published:** 2022-11-08

**Authors:** Zi-jian Zhou, Wei Ao, Li Fan, Shu-yi Zhang, Li-ping Cheng, Xiao-dong Xu, Jin-yu Zhao, Hui Zhang

**Affiliations:** 1 Lab of Modern Acoustics, Institute of Acoustics, Nanjing University, Nanjing, 210093 China; 2 Key Laboratory of Architectural Acoustic Environment of Anhui Higher Education Institutes, Anhui Jianzhu University, Hefei, 230601 China; 3 School of Mechanical Engineering, SouthEast University, Nanjing, 211189 China

**Keywords:** Applied physics, Condensed-matter physics, Physics

## Abstract

It is challenging to insulate sound transmission in low frequency-bands without blocking the air flow in a pipe. In this work, a small and light membrane-based cubic sound insulator is created to block acoustic waves in multiple low frequency-bands from 200 to 800 Hz in pipes. Due to distinct vibration modes of the membrane-type faces of the insulator and co-action of acoustic waves transmitting along different paths, large sound attenuation is achieved in multiple frequency-bands, and the maximum transmission loss reaches 25 dB. Furthermore, because the sound insulator with a deep subwavelength size is smaller than the cross-sectional area of the pipe, it does not block ventilation along the pipe.

## Introduction

Blocking sound transmission at a low frequency remains a challenging issue in acoustic because a sound insulator available at low frequencies requires a large size and/or a large mass according to the well-established mass law^1^. Although sound insulators with large sizes have been applied in industry^2^, it is required to miniaturize the insulators working at low frequencies to extend the application fields. An alternate method relies on active noise control^3^, which does not need a large or heavy sound insulator, while complex and expensive devices are inevitable. In these years, various artificial structures^4–11^ were presented for sound insulation and they exhibited extraordinary performance that cannot be obtained with natural materials or traditional acoustic devices. Thus, distinct methods for sound insulation were presented on the basis of these structures.

Bragg scattering^5^ and local resonance^7^ are considered to be the mechanisms of sound attenuation in artificial structures, in which sound insulators based on local resonance are more widely studied because of small bulks and varied structures. Membranes and/or plates were often adopted in resonance-based sound insulators because the resonance frequency of a membrane or plate could be easily decreased by decreasing the elasticity, and thus, sound attenuation at low frequencies was achieved by using an insulator with a small bulk and weight^6,8,9^.

It was presented that a membrane or plate provided dynamic negative density in the vicinity of the resonant frequency, which could be used to block the transmission of acoustic waves^10,12,13^. Additionally, attaching an extra mass on a membrane changed the resonance frequencies and vibration modes, which enhanced the sound attenuation^14–17^. Thus, membrane-type and plate-type structures were used to block sound in open space^14,16^ and in pipes^18–24^, and they exhibited potentials of application in HVAC (Heating, Ventilating and Air Conditioning) systems.

Although large sound attenuation was achieved, sound insulators with sealed structures were unavailable in a practical HVAC system because they completely block the air flow along the pipe. Thus, insulators with open structures were studied, which obstructed acoustic waves and did not block ventilation. Side structures were firstly used to insulate sound transmission along a pipe for ventilation, while the wall of the pipe must be opened when establishing the sound insulators^25,26^. Additionally, multiple folded Fabry–Perot resonators were set up on the inner wall of a pipe to produce sound attenuation at the resonant frequency^27^. Then, to reduce the bulk of the sound insulator, space-coiling^28,29^ and helical structures^30,31^ were adopted. These sound insulators produce attenuation within in a narrow frequency-band located at the resonant frequency of the structure. Meanwhile, membrane-type structures were also developed to simultaneously realize sound attenuation and air ventilation. A membrane with attached strips was used to replace a part of a pipe wall, which produces sound insulation at the resonant frequencies of distinct modes^32^. On the basis of the interaction of resonating field of four decorated membranes with the continuous sound field passing through a large orifice, a low-frequency and narrow-band acoustic filter was created^33^. Then, perfect absorption at a frequency below 500 Hz was obtained by utilizing coupled resonance of a membrane-type resonator and a decorated membrane^23^.

Although sound insulation at low-frequencies was realized using artificial structures on the basis of local resonance, working band width of the sound insulator was narrow due to the property of resonance. Numerical simulation demonstrated that a membrane-faced cuboid box (MFCB) could produce large sound attenuation in multiple frequency-bands in a low frequency range^34^. Thus, in this work, we create a small and light MFCB by 3D-print and establish an experimental apparatus to measure the performance of sound insulation of the MFCB. It is shown that the MFCB produces large sound attenuation in multiple low frequency-bands from 200 to 800 Hz, in which large losses over 21 dB are obtained at 211 Hz and 763 Hz. Distinct mechanisms are studied to explicate the sound insulation in the vicinity of four frequencies. The MFCB established in the pipe is smaller than the cross-sectional area of the pipe, and we design an experiment to study the influence of the MFCB on the air flow along the pipe, which demonstrates that the MFCB will not completely block ventilation along the pipe. Furthermore, we adopt a string of MFCBs to enhance sound attenuation, which further increases the transmission losses and expands the frequency-bands of sound attenuation.

## Results

**Figure 1:**
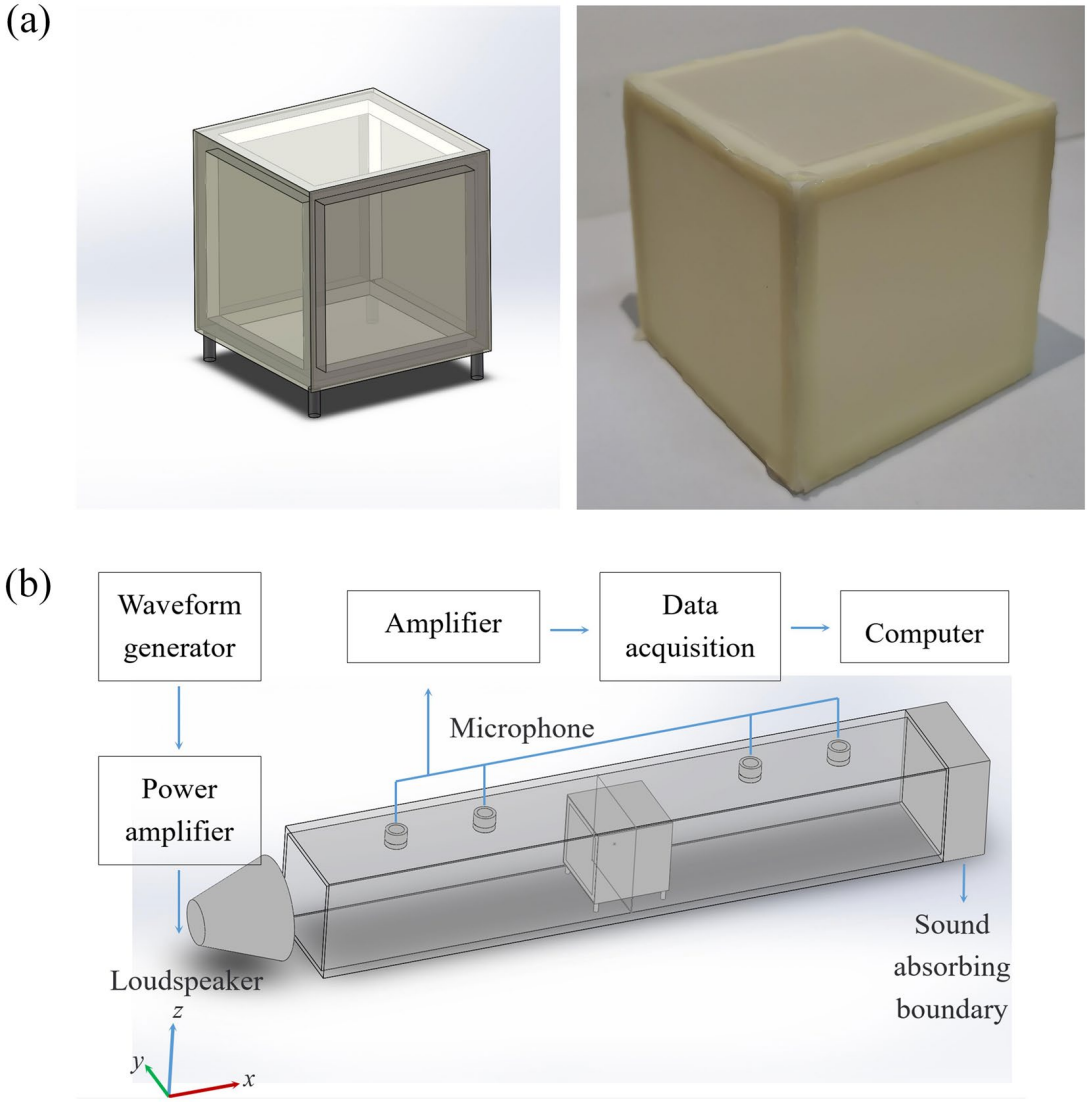
Experimental apparatus. (**a**) The model (left) and photo (right) of a MFCB. (**b**) Experimental apparatus for measuring the transmission loss induced by the MFCB. The distance between the adjacent microphones at the input end (or output end) is ${15}\;{\text{cm}}$ ${s}_{1}={s}_{2}=15\;\text{cm}$ and the distance between the microphone 2 (or 3) to the MFCB is 45 cm ${l}_{1}={l}_{2}=45\;\text{cm}$ (the figure is created using SOLIDWORKS 2016).

Figure 1a shows the model and photo of a MFCB and Fig. 1b shows the experimental apparatus established to measure the sound attenuation produced by the MFCB. The frame of the MFCB is made by 3D-print and the thickness of the frame is $0.5\;{\text{cm}}$. The frame is considered to be rigid to airborne sound waves. Elastic membranes are stretched and cover the frame, forming six square faces of the box, and thus, a MFCB is created (the details of the MFCB fabrication are shown in “Methods”: “Fabrication of a MFCB”). The weight of the MFCB is merely ${30}\;{\text{g}}$. The MFCB is supported in the middle of the pipe by four small brackets. The side lengths of the MFCB and the pipe are ${7}\;{\text{cm}}$ and ${9}\;{\text{cm}}$, respectively. Then, the MFCB does not completely block the pipe.

**Figure 2:**
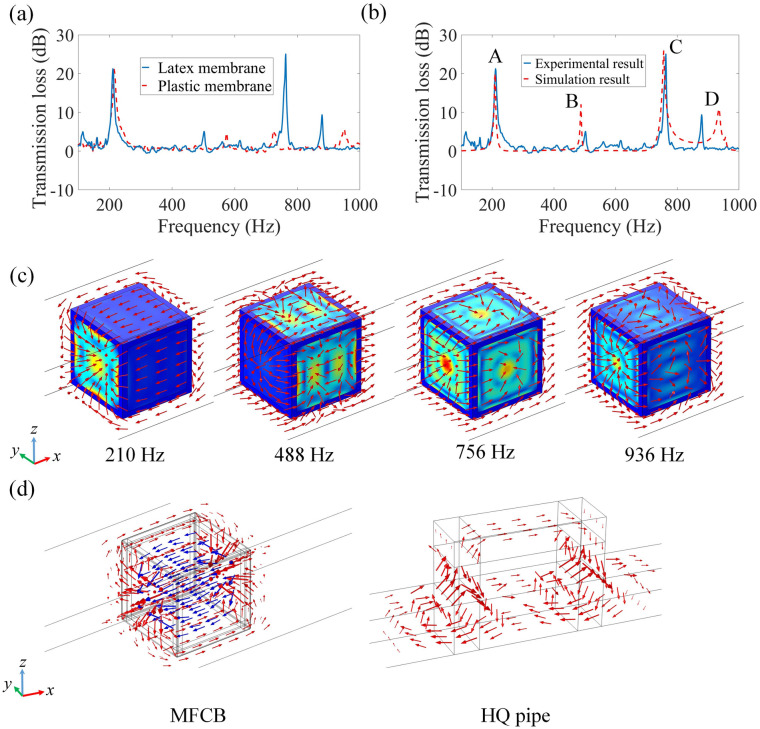
Transmission losses induced by the MFCB and the mechanism. (**a**) Measured transmission losses induced by the MFCBs created with two types of membranes, plastic and latex. (**b**) Comparison between the simulated and measured transmission losses induced by a MFCB based on latex membranes. (**c**) Vibration modes of the faces of the MFCB and acoustic intensities (red arrows) around the MFCB obtained at four sound attenuation peaks. (**d**) Comparison of acoustic intensities between the performance of a MFCB (Blue and red arrows indicate acoustic intensities in and around the MFCB, respectively.) and that of a HQ pipe (the figure is created using MATLAB 2016 and COMSOL 5.5).

Two types of membranes, plastic and latex membranes, are adopted in the experiments and the measured transmission losses using four-microphone method^35^ (see “Methods”: “Experimental apparatus for measuring the transmission loss”) are compared in Fig. 2a. It is observed that the MFCB using plastic membranes induces a large sound attenuation of 20 dB at 214 Hz, and meanwhile, approximate 5 dB transmission losses are obtained at frequencies of 574 Hz, 724 Hz, and 949 Hz. For the MFCB adopting latex membranes, large transmission losses of 21 and 25 dB are obtained at 211 Hz and 763 Hz, respectively, and two lower attenuation peaks arise at 502 Hz and 879 Hz. It is observed that the corresponding wavelengths at the lowest frequency 211 Hz of sound attenuation is 1.58 m, which is 22 times of side length of the MFCB. Thus, the MFCB with the size of deep sub-wavelength produces considerably large sound insulation at low frequencies.

The experimental results demonstrate that the MFCB with latex membranes exhibits better performance in sound insulation. Thus, we adopt the latex membranes to create our MFCB, and the parameters of the membranes are an original Young’s modulus of $E = 1.2 \times 10^{6} \;{\text{Pa}}$, ${E}_{m}=2\times {10}^{10} \; \mathrm{Pa}$, a Poisson’s ratio of $v = 0.40$ ${v}_{m}=0.40$, a density of $\rho = 970\;\; \text{kg}/\text{m}^{3}$ ${\rho }_{m}=970 \; \text{kg}/\text{m}^{3}$ and a thickness of $h = 0.08\;{\text{mm}}$ ${h}_{m}=0.08 \; \text{mm}$.These parameters are measured by an AG–X Plus Series Dual Column Electromechanical Test Frame.

When the membrane is stretched and pasted on the frame of the MFCB, it is challenging to obtain totally identical and even pre-stresses in the whole membrane and in different directions (The membrane locates in X–Y plane and Z is the normal direction) and the pre-stresses in distinct directions cannot be accurately measured. Thus, the simulated transmission loss induced by the MFCB on the basis of the pre-stresses in a stretched membrane deviate from the measured result (see part 3 of the Supplementary Materials for details). Thus, to accurately evaluate the performance the MFCB, we directly measure the equivalent Young's modulus^36^ $E_{eff}$ of the face by using an alternative method (see “Methods”: “Measurement of the equivalent Young's modulus of the MFCB face”). Thus, in the theoretical calculation and simulation, we adopt the measured equivalent Young's modulus of $E_{eff}  = 4.9 \times 10^{10} \;{\text{Pa}}$. Furthermore, the mechanical loss in the membrane is indicated by the ratio $\eta_{s}$ of the imaginary part to the real part of the Young's modulus, which is measured to be $\eta_{s} = 0.011$.

## Discussion

### Mechanisms for sound insulation in multiple frequency-bands

To study the mechanisms of the sound insulation in multiple frequency-bands, we simulate the performance of the MFCB based on latex membranes using COMSOL software. As shown in Fig. 2b, the simulated transmission loss exhibits four peaks at 210 Hz (A), 488 Hz (B), 756 Hz (C) and 936 Hz (D). Furthermore, Fig. 2c shows the vibration modes of the six faces of the MFCB and the acoustic intensities (red arrows) around the MFCB, which demonstrate that the transmission losses in multiple frequency-bands are induced by distinct vibration modes of the MFCB. It is noted that the vibrations of the six faces are determined by the symmetry of the MFCB. When the MFCB is established in a pipe, it exhibits rotational symmetry along the direction of the sound propagation (x-axis). Rotating the MFCB by ${\pi/{2}}$ does not change the performance and the acoustic fields in the pipe. Thus, the vibration modes of the four lateral faces parallel with the transmission direction (x-axis) of acoustic waves are identical. Meanwhile, the vibration modes of the front and rear faces (normal faces) perpendicular to the x-axis must satisfy ${\pi/{2}}$ rotational symmetry. Therefore, the vibration of the normal faces resembles to monopole, while a bipolar mode cannot be excited.

As shown in Fig. 2c, at the first sound attenuation peak A, both normal faces vibrate at the fundamental mode of a square membrane, while the four lateral faces marginally vibrate. Additionally, the acoustic intensities in and around the MFCB form a close loop, and thus, the majority of the sound energy is trapped in or around the MFCB and cannot transmit along the pipe. This mechanism resembles to that of a Herschel-Quincke (HQ) pipe, in which sound attenuation is induced by the co-action of the acoustic waves transmitting along two paths. The analogy of the MFCB to a HQ pipe is shown in Fig. 2d, in which similar fields of acoustic intensities in both structures are demonstrated.

It was well-established that a HQ pipe produces large transmission losses at two series of frequencies determined by^37^:

1 $$ S_{M} \sin \left( {kL_{S} } \right) + S_{S} \sin \left( {kL_{M} } \right) = 0, $$

in which $k$ is the wave number, $L_{M}$ and $L_{S}$ are the lengths of the main pipe and side loop, respectively. $S_{M}$ and $S_{S}$ are the cross-sectional areas of the main pipe and side loop, respectively. Then the sound attenuation frequencies are expressed to be $k(L_{M} - L_{S} ) = \left( {2n - 1} \right)\pi$ and $k(L_{M} + L_{S} ) = 2n\pi$, where $n$ is an integer. According to the analogy in Fig. 2d, a box with a side length of $a = 7\;{\text{cm}}$ is approximately considered to be a HQ pipe with $L_{M} = 7\;{\text{cm}}$ and $L_{S} = 14\;{\text{cm}}$. However, according to the mechanism of a traditional HQ pipe, the MFCB cannot create around attenuation at a low frequency of 210 Hz. It was established that sound attenuation induced by a traditional HQ pipe originates from the interaction of two acoustic waves with different phases after they transmit along two paths. While in the MFCB, both normal faces induce extra phase shifts to the acoustic waves traveling through the box, and thus, the equation determining the sound attenuation frequencies is rewritten to be:

2 $$ S_{S} \left[ {\sin \left( {kL_{M} } \right)\left( {1 + Z_{n}^{2} } \right) - 2jZ_{n} \cos \left( {kL_{M} } \right)} \right] + S_{M} \sin \left( {kL_{S} } \right) = 0, $$

in which $Z_{n} = {{S_{S} Z_{MA} }/{\rho_{0} }}c_{0}$ is the normalized acoustic impedance of the membrane, with $\rho_{0}$ and $c_{0}$ as the density and acoustic velocity of air (see part 1 of the Supplementary Materials for detailed derivation). The impedance $Z_{MA}$ of the membrane can be calculated by $Z_{MA} = j\omega M_{MA} + {1/{j\omega C_{MA} }}$, in which $M_{MA} = 2.06{{\rho h}/{a^{2} }}$ and $C_{MA} = 3.73 \times 10^{ - 4} {{a^{6} }/D}$, with $D = {{E_{eff} h^{3} }/{12(1 - \nu^{2} )}}$^10,38^. Then, from Eq. (2), we can obtain two solutions of 204 Hz and 219 Hz. It is observed that both frequencies locate near to each other and result in the peak A with large sound attenuation at 210 Hz. Thus, due to the extra phase shifts induced by $Z_{n}$, the small MFCB with the side length $a = 7\;{\text{cm}}$ can produce large sound attenuation within a low frequency-band. For a traditional HQ pipe, sound attenuation at a low frequency of 210 Hz cannot be achieved with the same size. Furthermore, the fundamental resonant frequency of the membrane is calculated to be 211 Hz, which locates near to the solutions of Eq. (2), 204 Hz and 219 Hz. Thus, it is demonstrated that the membranes dominate in the sound attenuation of peak A.

From Fig. 2c, it is observed that the second sound attenuation peak B is related to the resonance of the four lateral faces of the MFCB, and thus, a low peak B is obtained due to weak influences of the lateral faces on sound transmission. The resonance frequencies of the square faces can be expressed by $\omega a^{2} \sqrt {{\rho/D}} = \lambda$^39^, where $\omega$ is the circular frequency and $\lambda$ is a constant. It can be calculated that the frequency of the second resonant mode is 429 Hz with $\lambda = 73.42$^39^, which is near to the frequency 488 Hz of peak B. As shown in Fig. 2c, the sound attenuation peak C located at 756 Hz is related to the resonance of the same mode in the six MFCB faces, while the vibration in the normal faces is much more intensive than that in the lateral faces. The resonant frequency of this mode is calculated to be 777 Hz with $\lambda = 132.18$^39^. For both peaks A and C, intensive resonance arises in the normal faces, which results in large sound attenuation. For the peak D, the vibration mode of both normal faces differs from and that of the four lateral faces, and thus, the peak D is induced by hybridization of different resonant modes.

The differences between the simulated and measured transmission losses in Fig. 2b are primarily related to three factors. First, in the experiment, the MFCB is supported by four small brackets, which influence the symmetry in the four lateral faces. Second, the stress in the whole membrane is not even in the X and Y directions when it is stretched by hands. Finally, the six faces of a MFCB are not totally identical because it is challenging to evenly and identically exert stresses on the six faces of a MFCB. As shown in Fig. 2b, the measured peaks B and D derivate from the simulated ones. It can be observed in Fig. 2c, for peaks B and D, the four lateral faces exhibit similar vibration mode, and thus, nonsymmetrical lateral faces result in deviation of the sound attenuation peaks.

### Performance of multiple MFCBs

Although one MFCB can produce sound attenuation at multiple frequency-bands, these frequency-bands are narrow because they are related to the resonance of the MFCB faces. Then, we use multiple MFCBs to enhance sound insulation and expand the frequency-bands.

**Figure 3:**
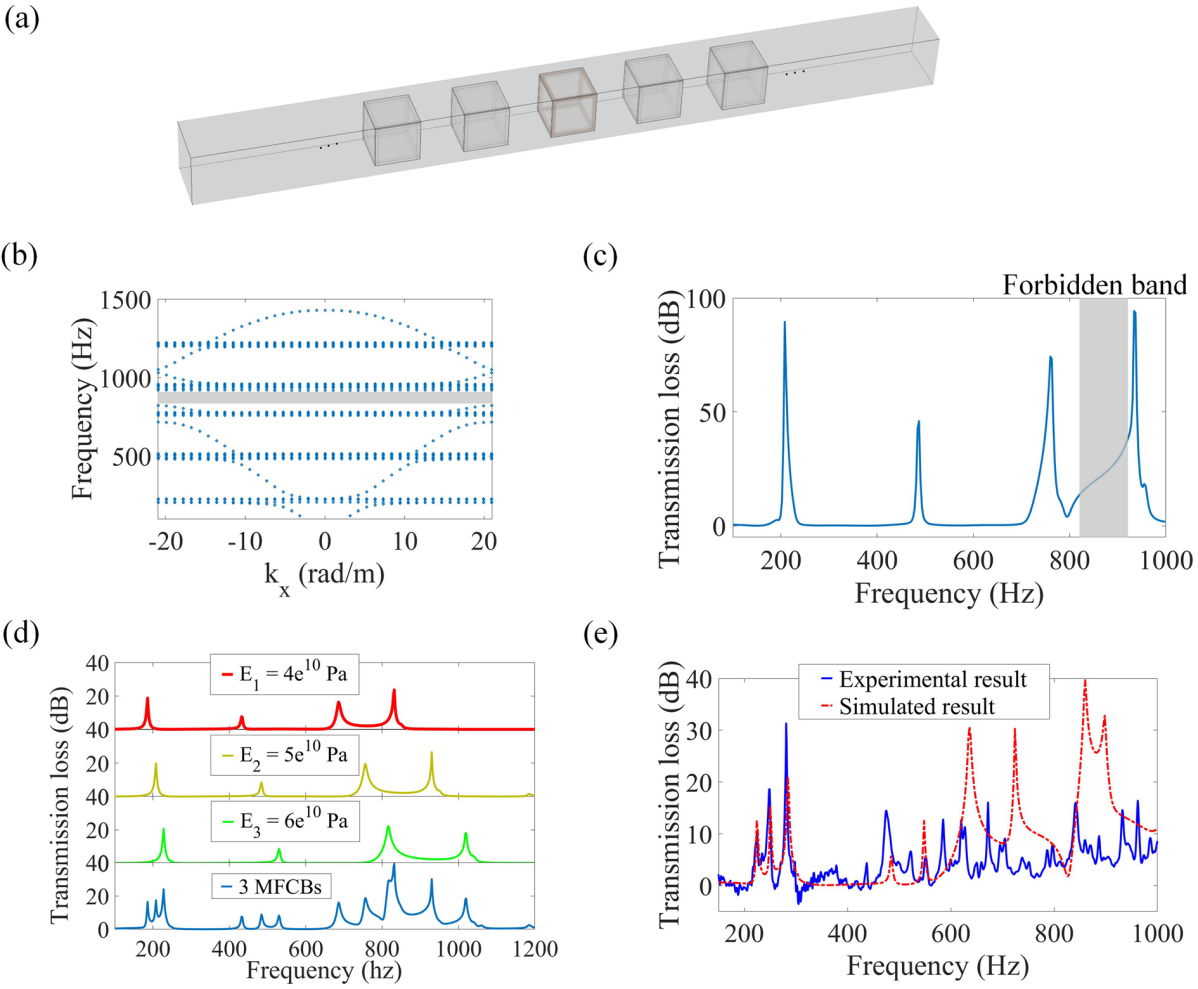
Performance of multiple MFCBs. (**a**) A string of MFCBs established in a pipe. (**b**) The dispersion of a periodic structure consisting of a string of MFCBs. The lattice constant is $d = 15\;{\text{cm}}$ and the equivalent Young's modii of the MFCBs are identical, which are $E = 4.9 \times 10^{10} \;{\text{Pa}}$. (**c**) Simulated transmission losses induced by a string of five MFCBs with identical parameters. (**d**) Simulated transmission losses induced by three MFCBs with different Young's moduli. (**e**) Comparing between simulated and measured transmission losses induced by three MFCBs with different Young's modii of $E_{1} = 6.1 \times 10^{10} \;{\text{Pa}}$, $E_{2} = 7.1 \times 10^{10} \;{\text{Pa}}$ and $E_{3} = 8.9 \times 10^{10} \;{\text{Pa}}$ (the figure is created using MATLAB 2016 and COMSOL 5.5).

As shown in Fig. 3a, multiple MFCBs are established along a pipe. Provided that the parameters of these MFCBs are identical, the string of MFCBs is considered to be a phononic crystal. Figure 3b shows the dispersion of the periodical structure with a lattice constant of $d = 15\;{\text{cm}}$, in which the parameters of each MFCB are the same as those used in the experiments. A forbidden band from 820 to 920 Hz is observed in Fig. 3b, which is induced by Bragg scattering. Additionally, it can be observed that four flat bands arise below 1 kHz, which are in agreement with the four sound attenuation peaks. It was demonstrated that these flat bands are related to local resonance^40^, which is consistent with the mechanisms for the sound insulation peaks. The transmission loss induced by the periodically established five MFCBs is shown in Fig. 3c, which shows that the sound attenuation frequencies are the same as those obtained with one single MFCB. Due to the superposition of the performance of multiple MFCBs, the peaks are much higher than those shown in Fig. 2b. However, the widths of these peaks are not widened except for a forbidden band induced by Bragg scattering.

To expand the frequency-bands of sound attenuation, we adjust the parameters of each MFCB in the string. The frequency-bands of sound attenuation can be shifted by adjusting the parameters of the membranes in a MFCB (see part 2 of Supplementary Materials). Transmission loss obtained with three MFCBs is shown in Fig. 3d, in which the Young's modii of the faces in each MFCB are changed to be $E_{1} = 4 \times 10^{10} \;{\text{Pa}}$, $E_{2} = 5 \times 10^{10} \;{\text{Pa}}$ and $E_{3} = 6 \times 10^{10} \;{\text{Pa}}$. In this case, the string of MFCBs cannot be considered to be a periodical structure. In Fig. 3d, it is observed that the transmission loss is a superposition of the sound attenuation induced by each MFCB in the string. Thus, the peaks with high transmission losses are widened, and furthermore, the transmission losses within these bands are increased. To evaluate the performance of multiple MFCBs, we measure the transmission loss obtained with three MFCBs and compare it with the simulated result in Fig. 3e. It is shown that the measured sound attenuation peaks in the low-frequency range between 210 and 290 Hz are in good agreement with the simulates ones. While the peaks at high frequencies differ from the simulated results because it is challenging to accurately and evenly exert identical pre-stresses in the faces of the three MFCBs used in the experiments. Compared to the performance of one MFCB, the sound attenuation is considerably enhanced by the co-action of the multiple MFCBs, and wide-band sound insulation with larger transmission losses is achieved.

### Performance of the MFCB in air flow

**Figure 4:**
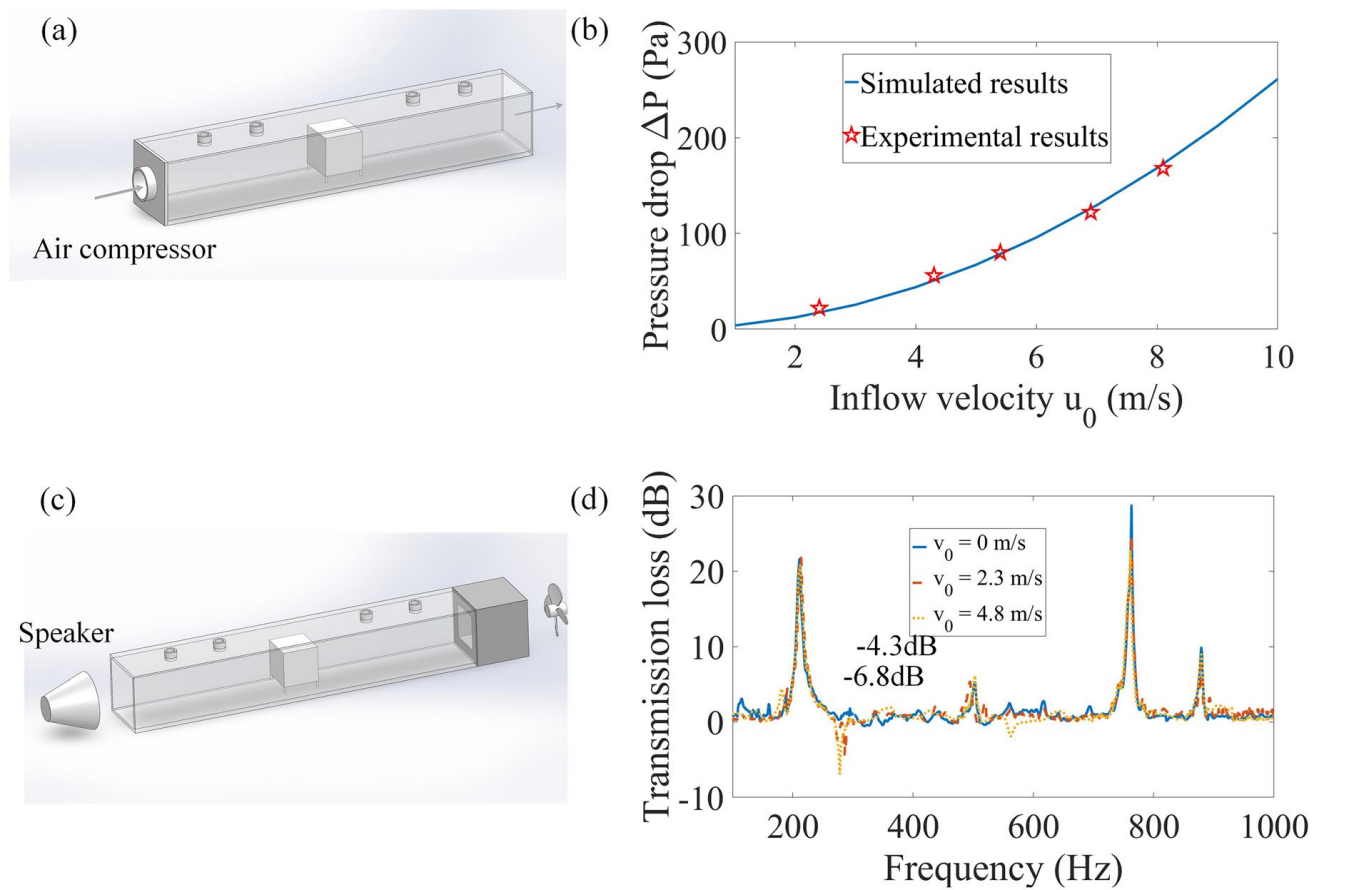
Influences of an air flow. (**a**) Experimental apparatus for evaluating the influence of the MFCB on the ventilation along a pipe. (**b**) Comparison of the measured and simulated results of the pressure drop induced by a MFCB with different input air flow velocities. (**c**) Experimental apparatus for measuring the transmission loss induced by a MFCB under an air flow. (**d**) Measured transmission losses with and without an air flow (the figure is created using SOLIDWORKS 2016, MATLAB 2016 and COMSOL 5.5).

Finally, we study the performance of our MFCB in an air flow. First, we establish an experimental apparatus shown in Fig. 4a to evaluate the influence of the MFCB on ventilation along a pipe. An air compressor is used to generate a constant air flow at the inlet of the pipe and the pressure drop $\Delta P$ induced by the MFCB is measured with a barometer. The results are shown in Fig. 4b. It is observed that the pressure drop $\Delta P$ increases with the input air flow velocity $u_{0}$, which is in good agreement with the simulated result obtained using the turbulent flow module in COMSOL. Furthermore, it was established that the pressure drop exerted by discontinuity in a pipe can be expressed by the following equation^41^:

3 $$ \Delta P = K_{e} \rho_{0} u_{0}^{2} , $$

in which $K_{e}$ is a dimensionless coefficient. It is observed from Fig. 4b that $\Delta P$ is quadratically related to $u_{0}$, which is consistent with Eq. (3). Then, in our experiment, we can obtain the coefficient $K_{e} = 2.02$ by fitting the simulated curve shown in Fig. 4b.

Furthermore, we study the influence of an air flow on sound insulation induced by the MFCB. Figure 4c shows the experimental apparatus and Fig. 4d indicates the transmission losses obtained with and without an air flow. It can be observed that the frequencies for the peaks of sound attenuation are not shifted by the air flow, while under an air flow, valleys arise in the transmission loss, which demonstrates that extra noise is induced by the MFCB. The noise is produced by the vibration of the light MFCB excited by the air flow. It is shown that under a flow velocity of $4.8\;{\text{m/s}}$ measured at the input of the pipe, extra noise is induced by the MFCB, which is 6.86 dB at 263 Hz and 1.91 dB at 562 Hz. Compared to the sound insulation, the induced noise is much lower and acceptable in a HVAC system for residence.

## Conclusion

To sum up, we present a structure of MFCB to block sound transmission along a pipe. Due to the interaction of two acoustic waves transmitting along two paths and the distinct vibration modes of the membrane-type faces of the MFCB, large sound attenuation is achieved in multiple frequency-bands between 200 to 800 Hz. Compared to the wavelength of the acoustic wave, the MFCB possesses a size of deep sub-wavelength. Additionally, the MFCB is smaller than the cross-sectional area of the pipe, which does not completely block the air flow along the pipe. Furthermore, by placing a string of the MFCBs in the pipe, the sound attenuation frequency-bands are expanded and the transmission losses are further increased. Thus, the MFCB exhibits potential application in sound insulation in pipes as HVAC systems.

## Methods

### Fabrication of a MFCB

The frame of the MFCB is made by 3D-print and the thickness of the frame is ${0}{\text{.5}}\;{\text{cm}}$. The frame is considered to be rigid to airborne sound waves. Square membranes with the side length of 8 cmare used to fabricate the faces of our MFCBs. First, we apply glue to the frame of a MFCB to fix the membrane. Then we exert a stress of $N = {2}0\;{\text{N/m}}$ in the X and Y directions of the membrane (Z direction is the normal direction) and paste it on the frame of the MFCB. Finally, we cut off the margin of the membrane. By repeating the process, we establish the membranes to six faces of the frame and create a MFCB.

### Experimental apparatus for measuring the transmission loss

Four-microphone method^35^ is applied to measure the sound attenuation induced by the MFCB. In this work, we study the sound attenuation induced by the MFCB in a low-frequency range below 1 kHz. The distance between both microphones (1, 2) at the input end [or output end (3,4)] is established to be 15 cm, which results in a critical frequency of 1.1 kHz. In this case, the critical frequency of the four-microphone method is not covered in the frequency range of experiments, from 100 Hz to 1 kHz. Additionally, the recorded data of 50 s is divided into 50 groups, and the data are averaged to reduce the influence of random noise.

### Measurement of the equivalent Young's modulus of the MFCB face

We evenly sprinkle powder with a mass of $M{ = }20\;{\text{g}}$ on the membrane and measure the average deformation $\Delta x_{avg} \approx 1.44 \times 10^{ - 4} \;{\text{m}}$ of the membrane with a laser rangefinder. Then, we can calculate the stiffness of the membrane to be $K \approx 1.36 \times 10^{3} \;{\text{N/m}}$. According to the relations as follows: $C_{a} = {{S_{M}^{2} } / K}$, $C_{a} = 3.73 \times 10^{ - 4} {{a^{6} } /D}$ and $D = {{E_{eff} h^{3} } /{12(1 - \nu^{2} )}}$^10,38^, where $a$ and $S_{M}$ are the side length and area of the face of a MFCB, we can calculate the equivalent Young's modulus $E_{eff} = 4.9 \times 10^{10} \;{\text{Pa}}$ of the MFCB face, which is adopted in the theoretical calculation and simulation. Additionally, the mechanical loss of the membrane is indicated by the ratio $\eta_{s}$ of the imaginary part to real part of the Young's modulus, which is measured to be $\eta_{s} = 0.011$ on the basis of Chinese national standard GB/T 18258-2000 “Damping materials-Testing method for damping properties”. Then, the Acoustic-Shell Interaction module in COMSOL is used to simulate the performance of the MFCB.

## Supplementary Information

Supplementary Information.
