# Supplementary material for: A low-frequency multiple-band sound insulator without blocking ventilation along a pipe
Source: Sci Rep. 2022 Nov 8;12:19034. doi: 10.1038/s41598-022-21673-8 (PMC9643527; doi:10.1038/s41598-022-21673-8)
Supplement: Supplementary file 1 — Supplementary Information. [file 41598_2022_21673_MOESM1_ESM.docx]

**A low-frequency multiple-band sound insulator without blocking ventilation along a pipe - Supplementary materials**

Zi-jian Zhou^1^, Wei Ao^1^, Li Fan^1^*, Shu-yi Zhang^1^*, Li-ping Cheng^1^, Xiao-dong Xu^1^, Jin-yu Zhao^2^ and Hui Zhang^1,3^

**1. Derivation of Eq. (2) in the main text**

For a traditional HQ pipe, the transfer matrices of the main pipe $\mathbf{T}$and side loop $\mathbf{S}$ are expressed to be:

$\mathbf{T}=\left[\begin{matrix} e^{-jkL_{M}} & 0 \\ 0 & e^{jkL_{M}} \end{matrix}\right]\text{;} \text{S}=\left[\begin{matrix} e^{-jkL_{S}} & 0 \\ 0 & e^{jkL_{S}} \end{matrix}\right]$, (S1)

in which $k$ is the wave number, $L_{M}$ and $L_{S}$ are the lengths of the main pipe and side loop, respectively. Then, we establish two membranes with the acoustic impedance $Z_{MA}$ and area $S_{M}$ in the main pipe at $x=0$ and $x=L_{S}$. According to the boundary conditions on both sides of the membrane, $v_{in}=v_{out}$ and $p_{in}-p_{out}=S_{M}v_{in}Z_{MA}$, where $v_{in}\left(p_{in}\right)$ and $v_{out}\left(p_{out}\right)$ indicate the particle velocities (acoustic pressures) on the left and right sides of the membrane, respectively, we can obtain the relation between the input and output acoustic pressures，$p_{in}$ and $p_{in}$, on both sides of the membrane as follows:

$\left[\begin{matrix} p_{out}^{+} \\ p_{out}^{-} \end{matrix}\right]=\left[\begin{matrix} 1-\frac{S_{M}Z_{MA}}{2\rho_{0}c_{0}} & \frac{S_{M}Z_{MA}}{2\rho_{0}c_{0}} \\ -\frac{S_{M}Z_{MA}}{2\rho_{0}c_{0}} & 1+\frac{S_{M}Z_{MA}}{2\rho_{0}c_{0}} \end{matrix}\right]\left[\begin{matrix} p_{in}^{+} \\ p_{in}^{-} \end{matrix}\right]=\mathbf{T}_{p}\left[\begin{matrix} p_{in}^{+} \\ p_{in}^{-} \end{matrix}\right]$, (S2)

in which the superscripts + and – indicate the incident and reflected acoustic pressures, respectively.

Then, we can obtain the transfer matrix for the main pipe with two membranes at $x=0$ and $x=L_{S}$, which is:

$\mathbf{T}_{M}=\mathbf{T}_{\mathbf{1}}\mathbf{TT}_{\mathbf{2}}=\left[\begin{matrix} T_{M11} & T_{M12} \\ T_{M21} & T_{M22} \end{matrix}\right]$, (S3)

where $\mathbf{T}_{\mathbf{1}}\mathbf{=T}_{\mathbf{2}}\mathbf{=T}_{\mathbf{p}}$.

Then, adopting the same method used in a traditional HQ pipe without a membrane, we can obtain the transfer matrix $\mathbf{T}_{r}=\left[T_{r11},T_{r12};T_{r21},T_{r22}\right]$ for a HQ pipe with two membranes, and the transmission loss induced by the HQ pipe is indicated by $TL=20\log_{10}\left|1/T_{r11}\right|$. The peaks of the transmission loss are determined by the zero points of the denominator of $1/T_{r11}$, which is expressed as follows:

$S_{M}\left|T_{M}\right|\alpha_{T}+S_{S}\left|S\right|\alpha_{S}\text{=}0$, (S4)

in which $\alpha_{T}=1/\left[\left(T_{M12}+T_{M22}\right)\text{-}\left(T_{M11}+T_{M21}\right)\right]$ and $\alpha_{S}=1/\left[\left(S_{12}+S_{22}\right)\text{-}\left(S_{11}+S_{21}\right)\right]$.$S_{M}$ and $S_{S}$ indicate the cross-sectional areas of the main pipe and side loop, respectively. Then, solving Eq. (S4), we obtain the equation determining the frequencies for the sound attenuation peaks:

$S_{S}\left[\sin\left(kL_{M}\right)\left(1+Z_{n}^{2}\right)-2jZ_{n}\cos\left(kL_{M}\right)\right]+S_{M}\sin\left(kL_{S}\right)=0$, (S5)

in which $Z_{n}=S_{M}Z_{MA}/\rho_{0}c_{0}$ is the normalized acoustic impedance of the membrane.

1. **Influences of the parameters of the membrane on sound attenuation induced by a MFCB**

The frequency-bands of sound attenuation induced by a MFCB can be shifted by adjusting the parameters of the faces. As shown in Fig. S1a, the four attenuation peaks exhibit blue shifts with the increase of the Young’s modulus $E$ from $5\times10^{10} \text{Pa}$ to $7\times10^{10} \text{Pa}$. Moreover, as indicated in Fig. S1b, increase of the density $\rho$ of the membrane results in red shifts of sound attenuation frequency-bands. The performance of the MFCB is greatly related to the resonant frequency of the membranes. Increasing
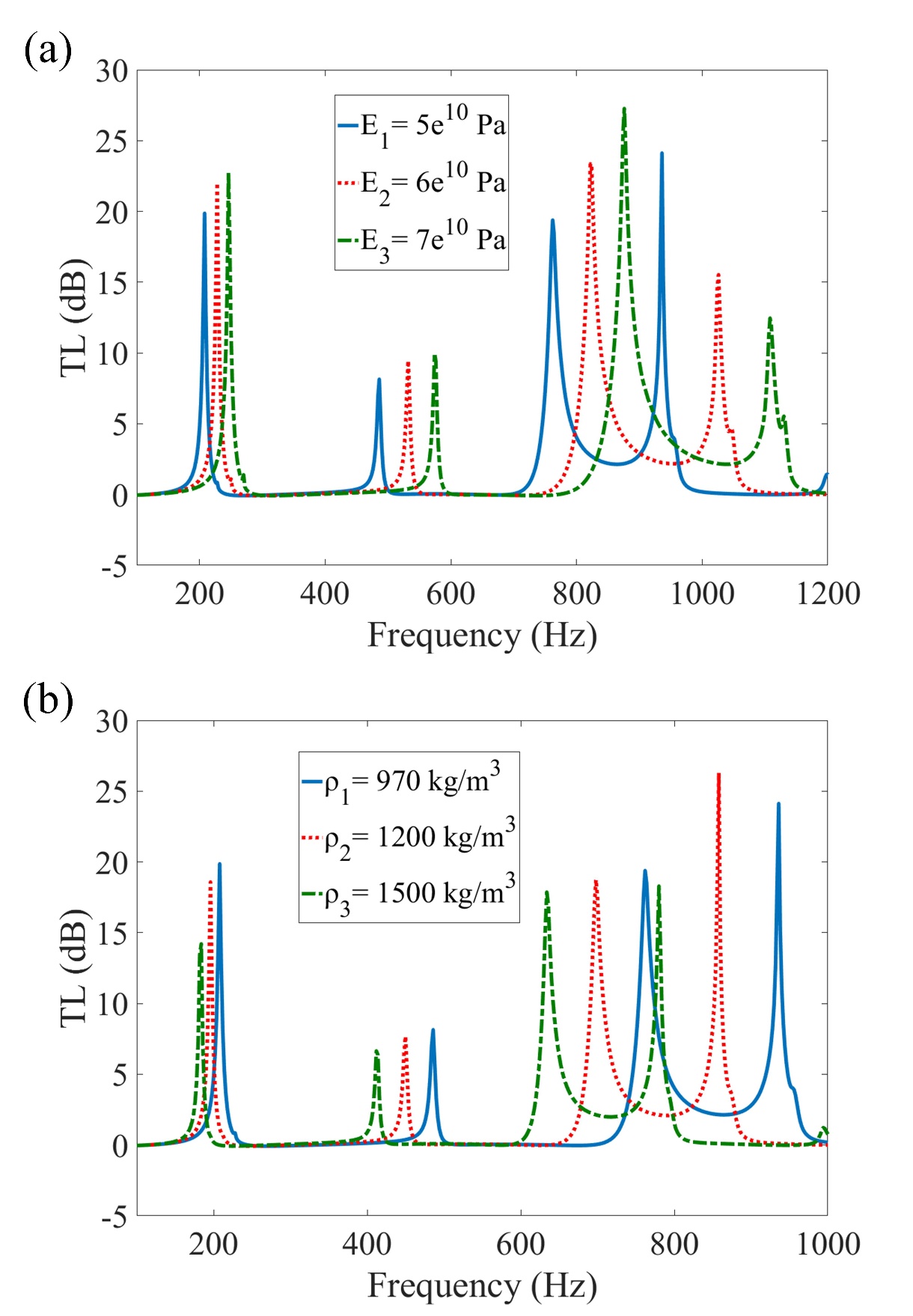


Fig. S1. (a, b) Influences of the (a) Young’s modulus and (b) density of the membrane on the frequency-bands of sound insulation.

$E$ of the membrane results in blue shifts of the eigenfrequencies and produces higher sound attenuation frequency-bands. While a larger density $\rho$ of the membrane results in lower resonance frequencies and produces red shifts of frequency-bands for sound insulation.

1. **Simulation models for the MFCB**

Generally, the faces of the MFCB are considered to be stretched membranes with pre-stresses. In the experiments, we use an AG-X Plus Series Dual Column Electromechanical Test Frame to measure the pre-stress in the membrane, which is$N=20N/m$. However, the AG-X can merely be available to measure the pre-stress exerted on the membrane in one direction, while the membrane is stretched in x- and y-directions when it is pasted to the frame of the MFCB. Furthermore, after the membrane is pasted to the frame, the pre-stresses in the membrane shift from the original value measured by AG-X. Additionally, the original Young's modulus $E=1.2\times10^{6}Pa$ of the membrane is measured on the basis of the ratio of the uniaxial tension to cross-sectional area, which is obtained using AG-X. When the membrane is pasted on the frame, biaxial pre-stresses arise in the membrane, which makes the membrane thinner and results in shift of the original Young's modulus. As a result, the simulated transmission loss on the basis of this model deviates from the measured result, as shown in Fig. S2. It is observed that the lowest peak of the simulated transmission loss is in agreement with that in the measured result, while the simulated peaks at high frequencies deviates from those obtained in experiments.

Thus, we use an alternative model to simulate the performance of the MFCB, in which an equivalent Young’s modulus is used to simulate the performance of the MFCB. In this case, because the equivalent Young’s modulus of the face can be measured more accurately when the membrane is attached on the frame of the MFCB, the simulated transmission loss using this model is in better agreement with the measured one, as shown in Fig. S2.


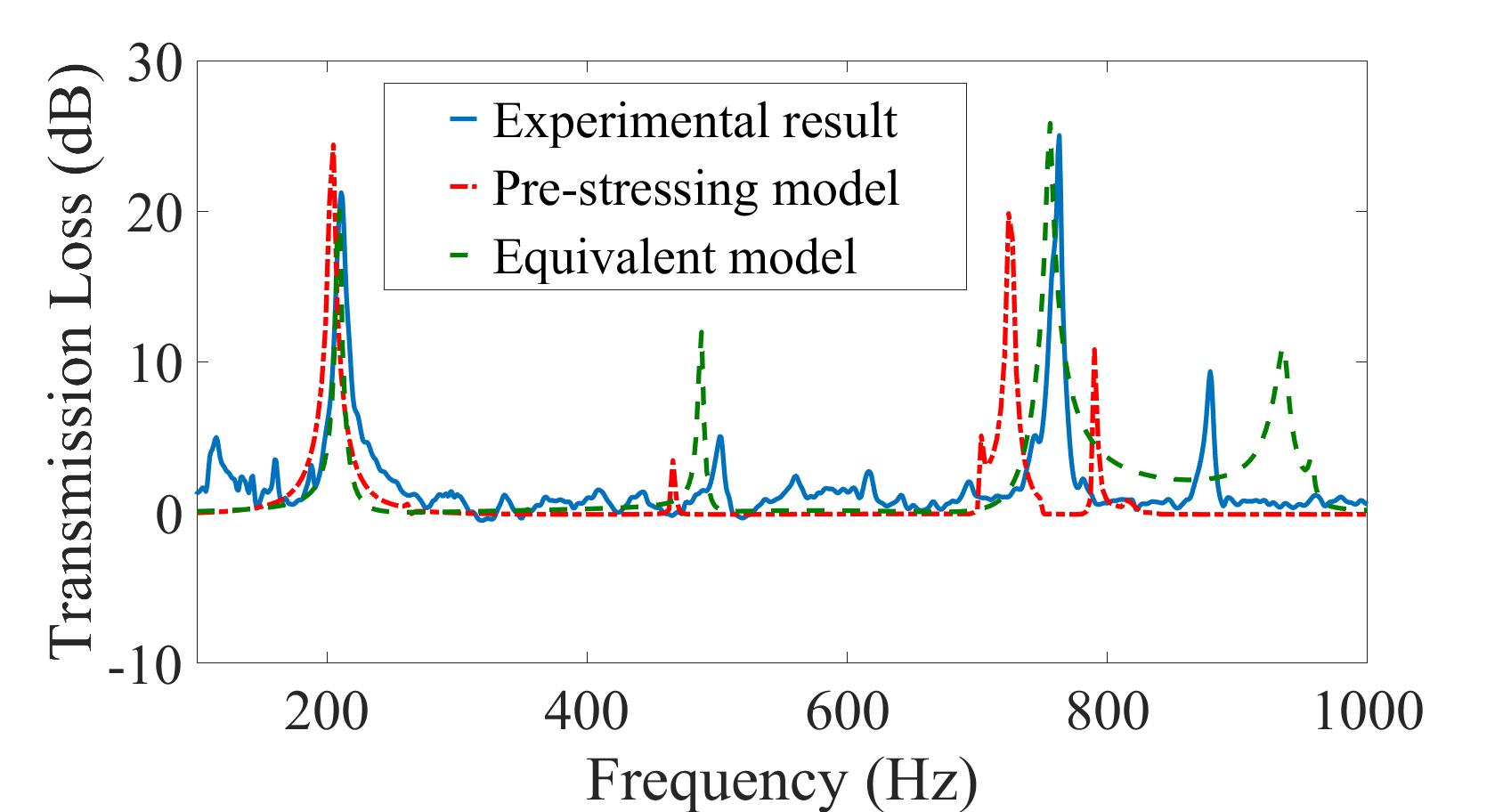


Fig. S2. Simulated and measured transmission losses.
